# PROLONGED DAILY FASTING TIMES ARE BEST WHEN COMBINED WITH CALORIE RESTRICTION FOR HEALTH AND SURVIVAL

**DOI:** 10.1093/geroni/igad104.0456

**Published:** 2023-12-21

**Authors:** Rafael de Cabo

**Affiliations:** National Institute on Aging, Baltimore, Maryland, United States

## Abstract

Emerging new evidence highlights the importance of prolonged daily fasting periods for the health and survival benefits of calorie restriction (CR) and time-restricted feeding (TRF) in male mice; however, little is known about the impact of these feeding regimens in females. Using a carousel feeder system that enables controlling the duration, amount, and timing of food availability, we show that mice on CR had a robust physiological response and decreased progression of malignant neoplasms and severity of inflammatory diseases in the lung compared to TRF. CR is associated with a unique serum metabolomics signature in overnight-fasted animals compared to TRF. The systemic impact of CR and TRF regimens measured from covariation of serum metabolites with energetics and healthspan metrics indicates that daytime (rest-phase) feeding with prolonged fasting periods initiated late in life confers greater benefits when combined with an imposed lower energy intake.

